# Prenatal Exposure to Maternal Depressed Mood and the *MTHFR* C677T Variant Affect *SLC6A4* Methylation in Infants at Birth

**DOI:** 10.1371/journal.pone.0012201

**Published:** 2010-08-16

**Authors:** Angela M. Devlin, Ursula Brain, Jehannine Austin, Tim F. Oberlander

**Affiliations:** 1 Department of Pediatrics, Child & Family Research Institute, University of British Columbia, Vancouver, Canada; 2 Early Human Experience Unit, Child & Family Research Institute, University of British Columbia, Vancouver, Canada; 3 Department of Psychiatry, Child & Family Research Institute, University of British Columbia, Vancouver, Canada; CNRS, France

## Abstract

**Background:**

Prenatal and early postnatal exposure to maternal depression may “program” childhood behavior via epigenetic processes such as DNA methylation. Methylenetetrahydro-folate reductase (MTHFR) is an important enzyme in the generation of methyl groups for DNA methylation. The common *MTHFR* C677T variant is associated with depression in men and non-pregnant women, and with global changes in DNA methylation. This study investigated the effect of maternal *MTHFR* C677T genotype on antenatal maternal mood, and their impact on the gene-specific methylation in pregnant women and their newborn infants. The methylation status of *SLC6A4*, which encodes the transmembrane serotonin transporter, and *BDNF*, which encodes brain derived neurotrophic factor, were assessed because of their potential role in behaviour.

**Methods/Principal Findings:**

Depressed mood was assessed by the Edinburgh Postnatal Depression Scale (EPDS) and the Hamilton Rating Scale for Depression (HAM-D) in women (n = 82, all taking folate) during the 2^nd^ and 3^rd^ trimesters of pregnancy. The methylation status of *SLC6A4* and *BDNF* were assessed in 3rd trimester maternal peripheral leukocytes and in umbilical cord leukocytes collected from their infants at birth. Women with the *MTHFR* 677TT genotype had greater 2^nd^ trimester depressed mood (p<0.05). Increased 2^nd^ trimester maternal depressed mood (EPDS scores) was associated with decreased maternal and infant *SLC6A4* promoter methylation (p<0.05), but had no effect on *BDNF* promoter methylation.

**Conclusions:**

These findings show that the *MTHFR* C677T variant is associated with greater depressed mood during pregnancy. We further showed that prenatal exposure to maternal depressed mood affects gene-specific DNA methylation patterns. These findings support the concept that alterations in epigenetic processes may contribute to developmental programming of behaviour by maternal depression.

## Introduction

Approximately, 15% of mothers experience mood disturbances during pregnancy and up to one third are treated with a serotonin reuptake inhibitor antidepressant (SRI) medication [1]. These two environmental factors may be among the earliest adverse life experiences that “programs” or (re-programs) the physiological, neuroendocrine and metabolic adaptations that underlie early human brain development, setting a course of health or illness that may last a life time. Increasing evidence points to the links between antenatal maternal depressed and anxious mood and risk for neurobehavioral disturbances during childhood [2], [3].

The molecular mechanisms underlying developmental programming are poorly understood but may involve the interplay between genetic and epigenetic processes, and prenatal environmental factors such as maternal mood. Epigenetic processes include DNA methylation and chromatin modifications (histone methylation, acetylation), patterns of which are inherited [4], [5] but are responsive to environmental shifts, such as stress, and are especially vulnerable during development [6]–[8]. For example, studies in a rodent model have shown that variations in early life experience (maternal care over the first week of life) is associated with decreased HPA stress responsivity in early infancy, and involves changes in the methylation status of the hippocampal glucocorticoid receptor (GR) gene (*Nr3c1*) and *Nr3c1* expression [6]. This phenomena has also been demonstrated in humans. We recently reported an association between exposure to increased 3^rd^ trimester maternal depressed mood and *NR3C1* promoter methylation in newborn infants, and HPA stress reactivity at 3 months [7], even when mothers had been treated with a selective serotonin reuptake inhibitor antidepressant. Furthermore, recent studies have shown methylation-silencing of rRNA and *NR3C1* expression in hippocampus from suicide victims with a history of child abuse [9], [10].

The neurochemical serotonin (5-HT) plays a critical link between early life experience and an increased risk for emotional disturbances in childhood [11]. Reduced 5-HT levels may increase a susceptibility for life time risk for depression, reflecting a “serotonergic vulnerability” [12]. A key regulator of 5-HT levels is the transmembrane serotonin transporter (5-HTT) that governs the reuptake of 5-HT and as such, determines the magnitude and duration of the 5-HT action. A 44 base pair insertion/deletion variant (referred to as 5-*HTTLPR*), in the promoter of the gene that encodes 5-HTT (*SLC6A4*), is believed to contribute to variations in 5-HTT expression, and as such, variations in 5-HTT-dependent 5-HT reuptake efficiency [13]–[15]. The 5-*HTTLPR* variant has been shown to influence vulnerability to the impact of early stressful life events [16], [17]. Furthermore, 5-HTT expression may also be regulated by epigenetic mechanisms. The methylation status of the *SLC6A4* promoter was shown to play a role in governing *SLC6A4* mRNA levels, however, this was dependent on the 5-*HTTLPR* genotype [18].

The objective of this study was to assess the effect of antenatal maternal depressed mood on the methylation status of *SLC6A4* and brain derived neurotrophic factor (*BDNF*) in pregnant women and their infants at birth. *SLC6A4* and *BDNF* were chosen as target genes because methylation plays a role in governing *SLC6A4* expression [8] and expression of *Bdnf* in a rat model was shown to be regulated by methylation and sensitive to early adverse life experience [8]. Several population studies have shown an association of the the gene for methylenetetrahydrofolate reductase (*MTHFR*), an enzyme required for folate metabolism and the generation of methyl groups [19], [20], with global changes in DNA methylation [21]–[23] and depressed mood and depressive disorders in non-pregnant populations [24]–[28]. As such, we further assessed the relationship of the *MTHFR* C677T variant with antenatal mood and S*LC6A4* and *BDNF* methylation status.

## Results

Maternal and neonatal demographic characteristics did not vary significantly with maternal *MTHFR* C677T genotype (Table 1). Genotype frequencies for the *MTHFR* C677T variant were 15.1% TT, 41.9% CT, and 43.0% CC (Table 2), similar to previous reports in non-pregnant women and men [19], [20], [28]. At 26 weeks gestation, women with the *MTHFR* 677TT genotype had significantly higher EPDS scores (F = 4.99; p = 0.009; ή^2^ = .11) compared to women with the *MTHFR* 677CT and 677CC genotypes (Table 2), controlling for serotonin reuptake inhibitor (SRI)-treatment. No association between *MTHFR* C677T genotype and maternal mood at 33 weeks was observed.

**Table 1:** Maternal *MTHFR* C677T genotype and demographic data of the pregnant women and their infants.

	Maternal *MTHFR* C677T Genotype		
	CC (n = 40)	CT (n = 36)	TT (n = 14)
**Maternal Characteristics**			
Maternal age at birth, years (SD)	32.4 (4.5)	33.4 (4.9)	31.4 (5.6)
Maternal education, years (SD)	16.8 (2.5)	16.5 (3.3)	15.6 (2.9)
Delivery type, % caesarian-section	36	31	36
SRI treated during pregnancy, %	38	48	57
Alcohol use - drinks during pregnancy, %			
None	60	47	100
1	7	11	0
2–10	22	28	0
>10	5	14	0
Tobacco Use, %	0	0	7
**Newborn Infant Characteristics**			
Prenatal SRI exposure, days (SD)	239 (52)	235 (71)	178 (85.6)
Birth weight, g (SD)	3469 (613)	3492 (476)	3523 (458)
Head Circumference, cm (SD)	34.8 (1.4)	34. 6 (1.3)	35.1 (1.3)
Length, cm (SD)	51.0 (3.15)	51.6 (2.6)	52.1 (2.5)
Gestational age at birth, weeks (SD)	40.0 (1.4)	39.3 (1.6)	39.8 (1.4)
Gender, % M / F	38/62	44/56	64/36
Apgar score at 1 minute (SD)	8.1 (1.5)	7.6 (1.5)	7.6 (1.5)
Apgar score at 5 minute (SD)	9.0 (0.46)	8.8 (0.78)	8.7(0.9)

**Table 2:** Influence of maternal *MTHFR* C677T and *BDNF* V66M genotypes on mood scores in the 2nd trimester of pregnancy^a^.

	*MTHFR* C677T Genotype (rs1801133)		
Mood Scores	CC (43.0%)	CT (41.9%)	TT (15.1%)
HAM-D total score	8.75±1.4	8.21±1.2	13.46±2.2
EPDS score	8.00±1.2	5.83±0.9	12.00±1.6^b^

Note: Values shown are means ± SE (standard error), *n* = 86 for *MTHFR* C677T.

a Maternal mood was assessed by the Edinburgh Postnatal Depression Scale (EPDS) [40] and the Hamilton Rating for Depression Scale (HAM-D) [39]. Effects of the *MTHFR* C677T variant on maternal mood scores was assessed by analysis of covariance (ANCOVA), with SRI-treatment as a covariate in the analysis.

b p<0.05 compared to *MTHFR* 677CC and 677CT genotype groups.

**Figure 1:**
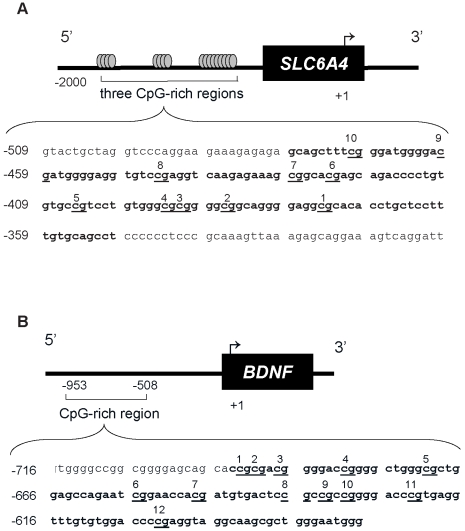
Schematic representation of the *SLC6A4* and *BDNF* promoters analyzed for methylation status. The portion analyzed by bisulfite pyrosequencing is shown in bold. The CpGs are underlined and numbered. Numbering of the gene sequence is relative to the transcriptional start site.

**Figure 2:**
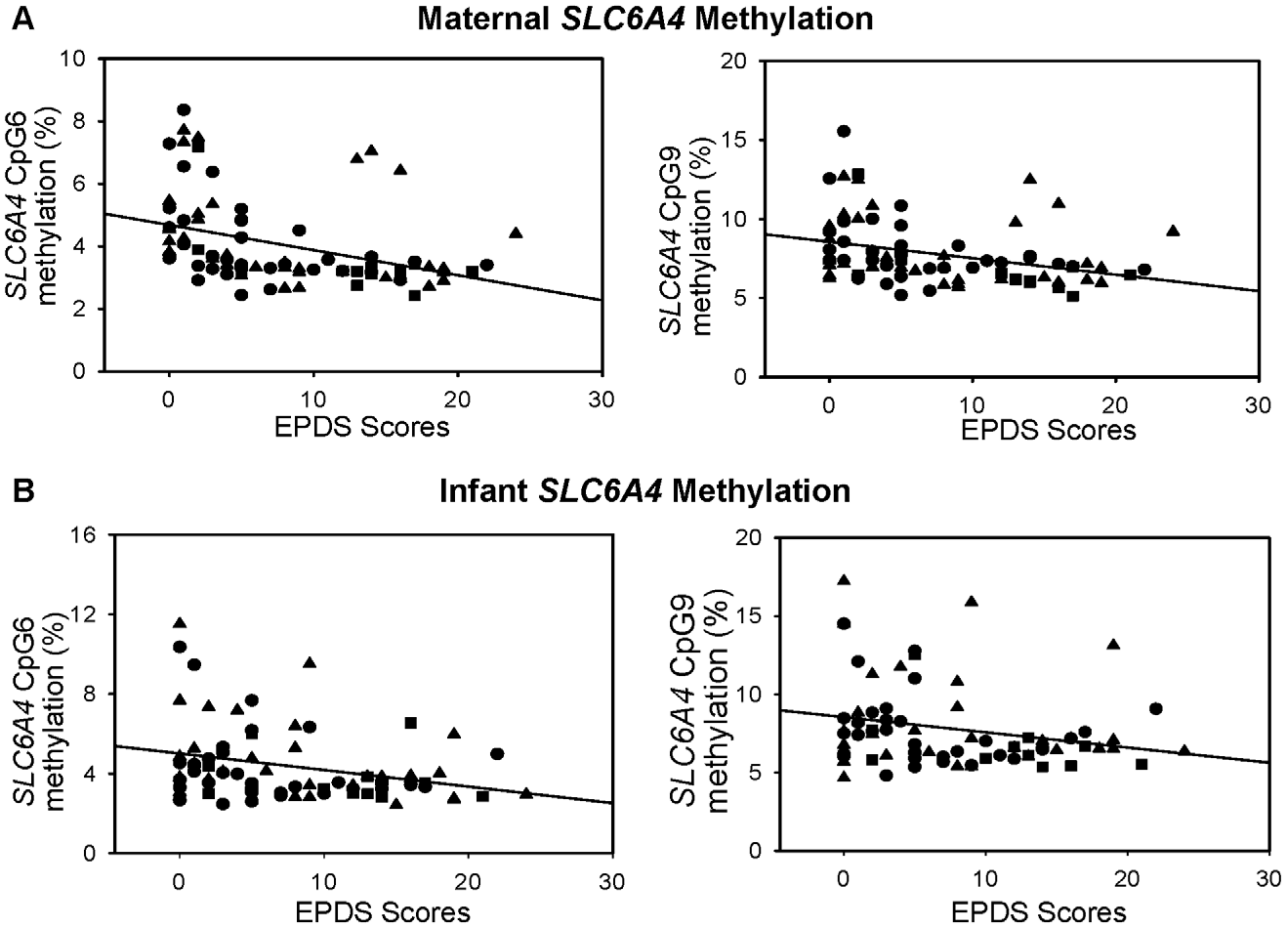
Relationship between *SLC6A4* promoter methylation status, maternal *MTHFR* C677T genotype, and pregnancy mood scores. The relationship between (**A**) maternal and (**B**) infant *SLC6A4* CpG 6 and CpG 9 methylation status and maternal EPDS scores (depressed mood scores) at 26 weeks gestation are shown. Points are plotted according to maternal *MTHFR* C677T genotype: solid triangle, CC; solid circle, CT; solid square, TT.

As shown in Figure 1, we assessed the methylation status of 10 CpGs in the *SLC6A4* promoter and 12 CpGs in the *BDNF* promoter. The methylation status of the *SLC6A4* promoter was significantly lower in mothers with increased depressed mood symptoms at 26 weeks gestation (p<0.05) (Table 3). Using a multivariate model, controlling for SRI exposure and *MTHFR* C677T genotype, the relationship between maternal depressed mood and lower methylation status was most evident for CpG sites 1,4,5,6,7 and 9 (sites 6 and 9 illustrated in Figure 2A), (F = 5.23, p = 0.024, ή^2^ = 0.065; F = 4.6, p = 0.034, ή^2^ = 0.058; F = 4.0, p = 0.050, ή^2^ = 0.050; F = 8.89, p = 0.004, ή^2^ = 0.104; F = 6.5, p = 0.013, ή^2^ = 0.078; F = 6.1, p = 0.015, ή^2^ = 0.075, respectively). Importantly, the methylation status of the *SLC6A4* promoter was unaffected by maternal *MTHFR* C677T genotype, SRI exposure, or mood at 33 weeks gestation. Maternal *BDNF* promoter methylation status was unaffected by maternal *MTHFR* C677T genotype, antenatal mood scores at 26 weeks and 33 weeks, or SRI exposure (Table 4).

**Table 3:** Maternal and infant *SLC6A4* promoter methylation status according to maternal *MTHFR* C677T genotype.

Maternal *SLC6A4* Methylation Status (% methylation)	Maternal *MTHFR* C677T Genotype			Infant *SLC6A4* Methylation Status (% methylation)	Maternal *MTHFR* C677T Genotype		
	CC (n = 35)	CT (n = 36)	TT (n = 12)		CC (n = 35)	CT (n = 36)	TT (n = 12)
CpG 1^a^	10.2±4.1	9.60±2.5	8.70±2.9	CpG 1	9.89±2.9	9.69±3.4	9.23±3.5
CpG 2	4.42±1.8	4.30±1.8	3.63±1.0	CpG 2	4.76±2.4	4.32±2.0	4.10±1.6
CpG 3^a^	7.38±2.1	7.22±2.1	6.16±1.5	CpG 3	7.80±2.6	7.40±2.6	7.94±4.2
CpG 4^a^	4.32±1.8	4.20±1.6	3.56±1.5	CpG 4	4.71±2.4	4.17±1.9	3.91±1.4
CpG 5^a^	7.35±1.9	7.58±1.9	6.44±1.4	CpG 5	7.62±2.2	7.26±1.9	7.54±3.6
CpG 6^a^	4.28±1.5	4.08±1.3	3.51±1.2	CpG 6^a^	4.61±2.1	4.31±1.8	3.83±1.3
CpG 7^a^	7.41±2.2	7.63±1.8	6.61±2.0	CpG 7	7.61±2.4	7.26±2.2	7.03±2.7
CpG 8^a^	5.44±1.3	5.45±1.0	4.87±1.0	CpG 8	5.77±1.5	5.69±1.4	4.99±1.6
CpG 9^a^	7.96±2.1	7.93±2.0	6.82±2.0	CpG 9^a^	8.31±3.2	7.66±2.2	6.82±2.0
CpG 10	8.39±1.7	8.50±1.3	8.36±1.2	CpG 10	10.18±2.0	10.00±1.6	9.40±2.0

Note: Values shown are means ± SE (standard error).

a p<0.05, significant effect of maternal Edinburgh Postnatal Depression Scale (EPDS) score (maternal mood) at 26 weeks gestation, as determined by analysis of covariance.

**Table 4:** Maternal and infant *BDNF* promoter methylation status according to maternal *MTHFR* C677T genotype.

Maternal *BDNF* Methylation Status (% methylation)	Maternal *MTHFR* C677T Genotype			Infant *BDNF* Methylation Status (% methylation)	Maternal *MTHFR* C677T Genotype		
	CC (n = 35)	CT (n = 36)	TT (n = 12)		CC (n = 35)	CT (n = 36)	TT (n = 12)
CpG 1	2.78±0.9	2.92±0.8	2.69±0.8	CpG 1	2.65±0.9	2.55±0.6	2.48±0.4
CpG 2	2.56±1.0	2.64±0.9	2.29±0.3	CpG 2	2.18±0.9	2.08±0.7	1.80±0.2
CpG 3	5.95±3.6	5.74±2.7	4.7±0.6	CpG 3	5.39±2.8	5.13±2.8	4.44±0.9
CpG 4	7.24±3.9	7.61±3.7	6.09±0.9	CpG 4	6.82±3.9	6.37±3.6	5.40±0.9
CpG 5	4.82±1.6	4.86±1.3	4.13±0.5	CpG 5	4.20±1.6	4.07±1.2	3.64±0.5
CpG 6	5.20±1.6	5.56±1.4	4.63±0.6	CpG 6	4.67±1.6	4.35±1.3	3.85±0.2
CpG 7	2.45±0.8	2.62±0.7	2.20±0.4	CpG 7	2.19±0.7	2.02±0.4	1.87±0.2
CpG 8	8.12±2.9	7.69±1.5	6.92±0.7	CpG 8	7.25±2.1	6.79±1.2	6.29±0.6
CpG 9	4.32±1.2	4.16±1.0	3.55±0.6	CpG 9	3.81±1.2	3.63±0.8	3.33±0.4
CpG 10	3.63±1.2	3.79±1.1	3.23±0.7	CpG 10	3.59±1.5	3.13±1.0	2.9±0.5
CpG 11	4.83±1.8	4.93±1.4	4.31±0.9	CpG 11	4.73±1.9	4.37±1.1	4.05±0.6
CpG 12	7.83±2.9	7.64±1.9	6.70±1.0	CpG 12	7.49±3.7	6.88±1.9	6.23±0.81

Note: Values shown are means ± SE (standard error).

Similar to what we observed for maternal *SLC6A4* promoter methylation status, decreased *SLC6A4* promoter methylation status at CpG site 6 and 9 in newborns was associated with increased levels of maternal depressed mood symptoms during the second trimester (F = 5.0, p = 0.029, ή^2^ = 0.070; F = 4.410, p = 0.039, ή^2^ = 0.06, respectively) (Table 3 and Figure 2B). To address the potential for a heritable *SLC6A4* epigenotype in infants we assessed the relationship of maternal *SLC6A4* promoter methylation status to infant *SLC6A4* methylation status and found no significant relationship. Methylation status was not associated with maternal and infant *MTHFR* C677T genotype or prenatal SRI exposure. Methylation status of the infant *SLC6A4* promoter was unaffected by maternal mood score at 33 weeks gestation. Neonatal *BDNF* promoter methylation status was not associated with either maternal or neonatal *MTHFR* C677T genotype, antenatal maternal mood scores, or prenatal SRI exposure (Table 4).

## Discussion

As a first step towards delineating a role for epigenetic mechanisms in the programming of childhood behaviour by prenatal exposure to maternal depressed mood we assessed the effect of antenatal maternal depressed mood on the methylation status of *SLC6A4* and *BDNF* in pregnant women and their infants at birth. There are three main findings of this study. First we found that that 2^nd^ trimester maternal depressed mood symptoms scores are associated with maternal *MTHFR* C677T genotype, such that women with the *MTHFR* 677TT genotype have the greatest depressed mood symptoms. Second, we found that antenatal maternal mood is associated with maternal and neonatal *SLC6A4* promoter methylation status. In particular, increased maternal depressed mood symptoms in the 2^nd^ trimester are associated with lower maternal *SLC6A4* promoter methylation status, but not the *BDNF* promoter. Interestingly, these associations were not observed during the 3^rd^ trimester and maternal SRI treatment did not play a role in any of these relationships. Third, similar to what we observed in the pregnant women, *SLC6A4* promoter methylation status was also lower in newborn infants from mothers who reported higher depressed mood symptoms during the 2^nd^ trimester. This relationship was unrelated to maternal *SLC6A4* methylation status, and unaffected by maternal and infant *MTHFR* C677T genotype, or prenatal exposure to maternal SRI medication. Given the role for *MTHFR* in methyl metabolism, these findings suggest that disturbances in methyl metabolism, such as those associated with the *MTHFR* 677TT genotype [19], [21], may contribute to the pathology of depression during pregnancy. These findings further suggest that prenatal exposure to maternal depressed mood during the second trimester of pregnancy can alter gene-specific DNA methylation patterns in newborns, and thereby set-up, via epigenetic mechanisms, processes that alter *SLC6A4* expression that may have long-term consequences. Given we found the *MTHFR* C677T variant is associated with greater antenatal depressed mood symptoms in women and that the *SLC6A4* promoter methylation status in women and infants was affected by maternal mood our finding of no direct effect of the *MTHFR* C677T variant on *SLC6A4* promoter methylation status was unexpected. The reason behind this finding is unknown but may simply be the consequence of insufficient power to detect such an effect, given the small number of women with the *MTHFR* 677TT genotype (n = 14).

In this study we report that increased maternal depressed mood during the 2^nd^ trimester of pregnancy was associated with reduced methylation of the maternal and neonatal *SLC6A4* promoter region. Conceivably, such reduced methylation may lead to increased *SLC6A4* expression and availability of 5-HTT, and as such, result in increased 5-HT reuptake and lower intrasynaptic 5-HT. In the mature brain this might not have a noticeable impact, but in the developing brain such altered serotonergic tone may have long term effects on behavior [29]. Prior to the neurotransmitter role of 5-HT, it plays critical roles as a trophic factor modulating neuronal differentiation and growth, therefore it is conceivable that changes in 5-HT via altered levels of the serotonin transporter during critical periods of development alters brain function and increases vulnerability to affective disorders later in life [30]. Altered central 5-HT, possibly via changes in methylation of regulatory regions of *SLC6A4* affects 5-HT levels during fetal development and may have a long term impact on the developing brain that “programs” subsequent child emotional development [31]. This has been demonstrated in *Slc6a4*−/− mice, which have no 5-HTT, increased intrasynaptic 5-HT (analogous to the pharmacological effect of an SRI), and increased depressed and anxious behaviors in adulthood, suggesting long-term consequences associated with early altered 5-HT levels [31]. The association between maternal mood and *SLC6A4* methylation status may offer an insight into processes, beyond genetic variations in *SLC6A4* that alters serotonergic tone during development. Demonstrating an effect of altered neonatal *SLC6A4* methylation status on developmental outcomes will provide evidence of a functional relationship and long-term consequences of such a relationship. These studies remain to be determined.

The methylation status of the *SLC6A4* promoter in whole blood from pregnant women and newborn infants observed in this study were within the same range previously reported by others for the mean methylation status of the *SLC6A4* promoter in lymphoblast cell lines [18]. In the current study we used bisulfite pyrosequencing to analyze a 130 bp region of the *SLC6A4* promoter adjacent to exon 1a, and quantified the methylation status of 10 CpG sites. This region corresponds to a portion of the much larger region of the *SLC6A4* promoter analyzed in lympophoblast cell lines that quantified 81 CpG sites by traditional bisulfite sequencing [18]. In this prior study it was also shown that the methylation status of 4 of the 81 CpG sites correlated with *SLC6A4* mRNA levels. In the region we analyzed CpG 8 corresponds to one of these sites at bp 872. We do recognize that our analysis was conducted in a heterogeneous mixture of cell types (whole blood), which may confound our findings. *SLC6A4* is expressed predominantly by platelets, lymphocytes, and monocytes in blood but in the current study we were unable to assess blood cell-specific differences in *SLC6A4* methylation status.

Interestingly *BDNF* methylation status was not affected by antenatal maternal mood, *MTHFR* C677T genotype or SRI exposure. One study did show differential methylation of 4 CpG sites in the coding sequence around the *BDNF* V66M variant, with the M allele associated with less methylation in human frontal cortex postmortem brain tissue [32]. The *BDNF* 66M allele has been associated with depression in elderly subjects [33] and is associated with reduced hippocampal volume [34]. In the current study we found no effect of the V66M variant on maternal depressed mood scores and no effect of maternal *BDNF* V66M genotype on maternal or infant *BDNF* promoter methylation status (results not shown). Studies in a rat model have shown that exposure to adverse maternal care giving in the first postnatal week following birth is associated with differential methylation of the 5′ region of the *Bdnf* gene and changes in *Bdnf* mRNA expression in prefrontal cortex from adult rats and that this is transferred to the next generation [8]. It remains to be determined how well gene-specific DNA methylation patterns in blood cells correlate with gene-specific DNA methylation patterns in brain regions, such as the hippocampus and prefrontal cortex.

Several studies have reported an association between the *MTHFR* C677T variant and depression [24]–[28] but the role of *MTHFR* in the pathology of depression remains to be determined. One would expect that the metabolic changes associated with the *MTHFR* 677TT genotype, such as elevated plasma total homocysteine [19], [20], or changes in global DNA methylation [21]–[23], are contributing factors. However, the degree of these changes are most pronounced in the presence of low folate status [23], [35]. The folate status of the women in our current study was not evaluated, but given all the women were taking folic acid supplements and living in an environment with mandatory folic acid fortification of the food supply it is unlikely that any of the women in our study had poor folate status.

Taken together, our findings suggest there may be a three-way interaction between maternal *MTHFR* C677T genotype, maternal depressed mood during pregnancy, and gene-specific changes in DNA methylation patterns such that maternal *MTHFR* 677TT genotype may predispose women to mood disturbances during pregnancy, which in turn influences gene-specific DNA methylation patterns, such as that observed for *SLC6A4*. Decreased methylation of the *SLC6A4* promoter may result in increased *SLC6A4* expression and changes in central serotonergic tone that might contribute to “programming” infant and childhood behaviour. This association between antenatal maternal depressed mood and *SLC6A4* methylation status is a first step towards a more complete understanding of how early life experience, genotype, and epigenetic processes contribute to development. Further studies are required to to assess the effect of *MTHFR* C677T variant, maternal mood and changes in *SLC6A4* promoter methylation status on *SLC6A4* expression and its impact on infant behaviour.

## Materials and Methods

### Subjects

With approval from the University of British Columbia Research Ethics Board, Children's and Women's Health Centre of British Columbia Research Review Committee, and written informed consent, a cohort (n = 98) of mothers was recruited in their early second trimester as part of a study of the impact of prenatal SRI exposure on neonatal health [36], [37]. Of the original 98 mothers who completed a second trimester data collection, samples from 16 mothers and infants at delivery were not available for analysis (i.e. mothers withdrew for personal reasons prior to delivery, inadequate DNA yield, infant cord blood sample was not obtained at birth) leaving 49 maternal and infant samples that were not treated with SRI medications, and 33 samples that were treated with SRI medications. Mothers were only included in the study if they took no other serotoninergic medications or other psychotropic medications during their pregnancy. All mothers were taking folic acid (1 mg folate/day) during their pregnancies, either as a component of a prenatal vitamin supplement or on its own. Maternal blood (mid 3^rd^ trimester) and neonatal cord (venous) blood samples were obtained for genotyping and DNA methylation analysis.

### Maternal Mood Assessment

Prenatal maternal mood was assessed using clinician- (blinded to SRI-treatment group status) and self-rated measures at the time of study enrollment (approximately 26 weeks) and at 33 weeks gestation. Measures included the *Hamilton Rating Scale for Depression* (HAM-D), a 21-item clinician administered scale designed to assess the severity of depression [38]. The *Edinburgh Postnatal Depression Scale* (EPDS) is a 10 item, self-rated instrument used to assess symptoms of depressed mood in both pre and postnatal settings [39]. Higher scores on these scales indicate higher levels of depression in the patient.

### Genotyping

Genomic DNA was extracted from maternal and newborn leukocytes using the Flexigene DNA Blood Kit (Qiagen, Valencia, CA). The *MTHFR* C677T (rs1801133), and *BDNF* V66M (rs6265) variants were genotyped using TaqMan SNP genotyping assay reagents and a 7500 Real Time PCR System (Applied Biosystems) following the manufacturer's suggested protocol.

### Quantitative Analysis of Gene-Specific DNA methylation

The methylation status of CpG-rich regions in the *SLC6A4* and *BDNF* gene promoters (Figure 1) were quantified by bisulfite Pyrosequencing [40]. The region of *SLC6A4* analyzed was within the same region shown to be differentially methylated and associated with changes in *SLC6A4* mRNA expression [18], [41]. We analyzed a region of the *SLC6A4* promoter between −479 and −350, relative to the transcriptional start site, which contains 10 CpG sites and is adjacent to exon 1a [42]. For *BDNF* we analyzed a CpG-rich region of the promoter between −694 and −577, relative to the transcriptional start, which contains 12 CpG sites. The region of *BDNF* we analyzed for methylation status corresponds to an analogous region in rat *Bdnf*, which was shown to be differentially methylated and associated with *Bdnf* mRNA expression [8], [43].

Genomic DNA from leukocytes (1 μg) was bisulfite-treated using the EpiTect Bisulfite Kit (Qiagen) following the manufacturer's suggested protocol, and stored at −20°C until further analysis. A 130 bp fragment of the *SLC6A4* promoter (Fig. 1) and a 118 bp fragment of the *BDNF* promoter were amplified by PCR from bisulfite-treated DNA using HotStar Taq DNA Polymerase (Qiagen) and the following primers for *SLC6A4*: PMHSERTF, 5′-GTATTGTTAGG TTTTAGGAAGAAAGAGAGA-3′ and PMHSERTR, 5′-AAAAATCCTAACTTTCCTACTCT TTAACTT-3′; and for *BDNF*: PMHBDNFF, 5′-GTGGGGTAGGAGGGGAGTAGTAT-3′ and PMHBDNFR, 5′-AAATCCCCCAATCAACTCTCT-3′ (IDT Inc, Coralville, IA), with the reverse primer containing a biotin at the 5′ end. Cycling conditions were 94°C for 15 minutes followed by 50 cycles of 94°C for 1 minute, 60°C for 1 minute, and 72°C for 1 minute with a final extension of 10 minutes at 72°C. PCR products were purified and sequenced using a PyroMark MD System (Biotage, Foxboro, MA) following the manufacturer's suggested protocol and the following sequencing primers for *SLC6A4* and *BDNF*, respectively: PMHSERTS, 5′-AA ACTACACAAAAAAACAAAT-3′ and PMHBDNFS, 5′-GGTAGGAGGGGAGTAGTA-3′ (IDT). The percent methylation at each CpG site was quantified using the Pyro Q-CpG software, version 1.0.9 (Biotage).

### Statistical Analyses

The effects of the *MTHFR* C677T variant on maternal depressed mood scores was assessed by analysis of covariance (ANCOVA), with genotype as the independent variable and SRI-treatment as a covariate in the analysis. The effect of maternal and infant *MTHFR* C677T genotype on maternal and infant *SLC6A4* and *BDNF* promoter methylation status at specific CpG sites was assessed using multiple analyses of covariance (MANCOVA) models with maternal EPDS score (depressed mood score) and SRI-treatment as covariates. Effect sizes (eta squared) were also calculated. All analysis was conducted using SPSS, version 16.0 (SPSS Inc, Chicago, IL).
